# Household, psychosocial, and individual-level factors associated with fruit, vegetable, and fiber intake among low-income urban African American youth

**DOI:** 10.1186/s12889-016-3499-6

**Published:** 2016-08-24

**Authors:** Angela Cristina Bizzotto Trude, Anna Yevgenyevna Kharmats, Kristen Marie Hurley, Elizabeth Anderson Steeves, Sameera A. Talegawkar, Joel Gittelsohn

**Affiliations:** 1 The Johns Hopkins Bloomberg School of Public Health, Department of International Health, Global Obesity Prevention Center and Center for Human Nutrition, 615 N. Wolfe Street, Baltimore, MD 21205 USA; 2 Department of Nutrition, University of Tennessee, 1215 W. Cumberland Ave., Knoxville, TN 37996 USA; 3 Milken Institute School of Public Health, Department of Exercise and Nutrition Sciences, The George Washington University, 950 New Hampshire Ave, NW, Washington, DC 20052 USA

**Keywords:** Fruit and vegetable intake, Fiber, Youth, Food purchasing, Eating behavior

## Abstract

**Background:**

Childhood obesity, one of the greatest challenges to public health, disproportionately affects low-income urban minority populations. Fruits and vegetables (FV) are nutrient dense foods that may be inversely associated with excessive weight gain. We aimed to identify the individual characteristic, psychosocial, and household factors influencing FV and fiber consumption in low-income African-American (AA) youth in Baltimore, MD.

**Methods:**

Cross-sectional analysis of data collected from 285 low-income AA caregiver-youth (age range: 10–14 y) dyads participating in the baseline evaluation of the B’More Healthy Communities for Kids obesity prevention trial. The Kid's Block FFQ was used to estimate daily intakes of FV (including 100 % fruit juice) and dietary fiber. Questionnaires were used to assess household socio-demographics, caregiver and youth food purchasing and preparation behavior, and youth psychosocial information. Ordered logit regression analyses were conducted to examine psychosocial and food-related behavior associated with FV and dietary fiber intake (quartile of intake) controlling for youth age, sex, BMI percentile, total calorie intake and household income.

**Results:**

On average, youth consumed 1.5 ± 1.1 (M ± SD) servings of fruit, 1.8 ± 1.7 serving of vegetables, and 15.3 ± 10.9 g of fiber/day. There were no differences by gender, age or household income. Greater youth’s healthy eating intentions and self-efficacy scores were associated with greater odds ratio for higher intake of FV and fiber (Intention: OR_fruit_ 1.22; 95 % CI: 1.06–1.41, OR_vegetable_ 1.31; 1.15–1.51 and OR_fiber_ 1.46; 1.23–1.74, Self-efficacy: OR_fruit_ 1.07; 1.03–1.12, OR_vegetable_ 1.04; 1.01–1.09, OR_fiber_ 1.10; 1.04–1.16). Youth receiving free/low-cost breakfast were more than twice as likely to have higher fiber intake than those who did not receive free breakfast (OR 2.7; 1.10; 6.9). In addition, youth shopping more frequently at supermarkets were more likely to have greater vegetable and fiber intake (OR 1.26; 1.06–1.50; OR 1.28; 1.03–1.58, respectively). Also, youth with parents who shopped more frequently at fast-food stores had 7 % lower odds for higher vegetable intake (95 % CI: 0.88–0.99).

**Conclusion:**

In this study, both, youth and household factors were associated with youth FV and fiber intake, underscoring the need for a multi-level approach to increasing youths’ diet quality. These results will inform and shape an effective intervention program for improving youth dietary intakes.

**Electronic supplementary material:**

The online version of this article (doi:10.1186/s12889-016-3499-6) contains supplementary material, which is available to authorized users.

## Background

Childhood obesity is a significant public health problem [1, 2]. The diets of youth today, especially in low-income, minority urban populations, are often characterized by a high intake of refined carbohydrates, added sugars, fats, and salt due to high consumption of energy dense, processed foods [3]. Conversely, low intake of low-calorie, healthy promoting, fiber-rich fruits and vegetables (FV) may place youth at higher risk for obesity and chronic disease [4, 5].

Dietary fiber from whole grains, fruits, and vegetables is often associated with a higher diet quality and variety [6, 7] and is recommended by dietary guidelines for its health promotion characteristics [8]. Most youth in the U.S. do not achieve the recommended amount of fruit and vegetables. According to the Youth Risk Behavior Surveillance, in the U.S. only a third of the youth (10–24 years old) interviewed had consumed two or more servings of fruit per day, and only 15 % had eaten three or more servings of vegetables per day within the past week, with FV intake even lower among low-income and minority youth [9]. For example, African American (AA) youth consume fewer FV - 6.9 % for fruit and 11.3 % for vegetables - than White and Hispanic youth [9]. Additionally, a recent a review of the literature across 31 qualitative studies suggested that low-income families are more likely to have a lower intake of fruits and vegetables than high-income families [10].

According to the Social Cognitive Theory, psychosocial factors might influence eating behavior, as it has been theorized that the cognitive processes play an important role in the acquisition and retention of new behavior patterns [11]. Psychosocial factors such as self-efficacy, expectancy and food knowledge have been found to be associated with higher consumption of fruits and vegetables in youth [12]. Self-efficacy, as an indicator of confidence and decision-making about healthy eating, is the most commonly measured psychosocial construct and has been identified as an important predictor of fruit and vegetable intake [13–15]. However, other studies have not found consistent association between these psychosocial factors and fruit and vegetable intake [16, 17].

Several household level factors are known to influence youth’s consumption of FV. Parents play a critical role in influencing youths’ eating behavior by controlling their food environment and acting as role models for eating behaviors [18]. In a previous study, eating meals prepared at home and involving youth in the cooking process were identified as household determinants of fruit and vegetable consumption [19]. Recently, one study documented that American families do not spend as much time cooking and preparing meals as in the 1960s, due to an increase in eating food in restaurants and obtaining food from carryouts and other prepared food sources [20]. Due to the high availability of high energy-dense food and the low availability of fruit and vegetable in carryouts and fast-food sources, youth are exposed to low quality meals that increase the risk of diet-related chronic diseases [21–23].

Rates of consumption of fruit decrease from age 11 (boys: 44 %; girls: 49 % eat fruit daily) to 15 (boys: 32 %; girls: 34 %) [24]. Given the low-intake of fruit and vegetable during the adolescent period [9], it is important to identify risk factors to inform diet-related interventions and health programs among vulnerable populations. To our knowledge, few studies have examined the multi-level factors associated with fruit and vegetable intake among low-income, urban minority adolescent youth [25, 26].

This study aims to identify the multi-level factors influencing fruit and vegetable consumption in African-American (AA) youth in Baltimore, MD. The results will help to provide information to promote fruit and vegetable consumption in an ongoing multi-level intervention trial in Baltimore City [26]. We hypothesize that youth psychosocial factors (e.g. intentions to eat healthy food, self-efficacy, food knowledge, and outcome expectancy) and food behavior (e.g. youth food purchasing and food preparation behavior, and food assistance) and household-level food related behaviors (e.g. income, caregiver food purchasing and food preparation behavior, and food assistance) would be associated with youth’s intake of fruit, vegetable and fiber. Specifically, we examined:

1. The fruit, vegetable and fiber consumption patterns in a sample of low-income urban AA youth, and if these met the daily Dietary Guidelines for Americans recommendations.
2. The youth-level psychosocial and food-related behavior factors associated with fruit, vegetable, and fiber intake among low-income AA youth.
3. The household-level food-related behavior factors associated with fruit, vegetable, and fiber intake among low-income AA youth.

## Methods

### Study design

This is a cross-sectional analysis of baseline data from the B’more Healthy Communities for Kids (BHCK) study. BHCK is an ongoing intervention trial operating at multiple levels of the Baltimore City food system and focuses on childhood obesity prevention to improve access to and consumption of healthy foods in low-income Baltimore City neighborhoods among African-American youth [27]. Baseline data were collected from July 2013 to June 2014 [26].

### Participants

Participants in this study were youth actively recruited from 14 low-income, predominantly African American neighborhoods that were defined as food deserts [28]. A list of potential youth was created and screened for eligibility. Youth eligibility criteria included: 1) between the ages of 10 and 14 when recruited and consent from an adult caregiver; 2) residence within a mile and a half radius of the neighborhood recreation center; and 3) no intentions of moving within the next two years. Among those recruited and screened, 24 youth-caregiver dyads were interviewed in each neighborhood. In summary, we interviewed 285 youth and their caregivers living in low-income urban Baltimore City.

### Data collection

Data collectors were trained intensively on data collection tools. Training consisted of instruction on enrolling, consenting, general questionnaire techniques, and anthropometric measurement techniques. In-class practice, role-play and observation and feedback of data collectors from the lead investigator (JG) were used. Youth and caregivers received gift cards for interview participation. Informed assent and consent were gathered from the youth and caregiver, respectively. Following the interviews, data were checked for errors by the interviewer and a second research assistant. The data manager ensured that questionnaires were not missing pages or data, such as gender, birth date, etc. The data manager also provided data collectors with constructive feedback on completing the questionnaire during the course of data collection. All data collection questions and issues were handled on a weekly basis with the lead investigator and data manager. This study was approved by the Johns Hopkins Bloomberg School of Public Health Institutional Review Board (IRB No. 00004203).

### Measurements

#### Youth fruit, vegetable, and fiber intake

Two data collection instruments were used to interview youth – the Block Kids 2004 Food Frequency Questionnaire [29] (BKFFQ) and a Youth Impact Questionnaire (CIQ) [30]. The BKFFQ instrument is a semi-quantitative, validated questionnaire in adolescent populations [29, 31] that ascertains the frequency and consumption amount of 77 common food items in the previous week. The frequency of consumption ranges from ‘none’ to ‘every day’ , and quantities consumed are assessed with three to four categories related to food type. It contains foods identified by NHANES II commonly consumed by youth. Completed FFQs were analyzed by NutritionQuest (Berkley, California, USA) and estimates of food and nutrition intakes were generated for each youth. Daily fruit and vegetable intake were estimated in cup equivalent servings and dietary fiber was estimated in grams. Vegetable servings excluded potatoes and legumes, and fruit servings included 100 % fruit juice. The food groups for the database for the BKFFQ were developed using NHANES and the USDA’s My Pyramid Equivalents Database 2.0 (MPED). All foods and beverages reported in the NHANES 24 h recalls were assigned values in the MPED database. Most foods, including mixed dishes, contributed to more than one food group.

#### Youth-level psychosocial factors and food-related behaviors

The CIQ consisted of 79 questions and was used to collect information pertaining to youth food consumption, food preparation and food-purchasing habits, along with demographic measures and psychosocial factors related to healthy eating, including behavioral intentions, outcome expectancies, self-efficacy, and knowledge [30, 32].

#### Household socio-demographic and food-related behaviors

Household level information was assessed through the Adult Impact Questionnaire (AIQ) answered by the youth’s primary caregiver. This is a 176-item questionnaire and includes questions on demographics and household socioeconomic information (e.g. parental education, marital status, employment status, and household income, housing arrangement of the primary caregiver, and household participation in food assistance programs), as well as questions on food purchasing, and food preparation.

Both instruments – Adult Impact Questionnaire (AIQ) and Child Impact Questionnaire (CIQ) – were developed from similar instruments used in previous intervention trials and on the basis of formative research [33, 34]. The instrument was finalized after pilot testing with community members and conducting face validity on 15 randomly selected adult and child respondents [35]. Participants were interviewed in depth to verify the clarity of and their response to the questions. We used Cronbach’s alpha (described below) to calculate the reliability of the scores.

#### Anthropometry

Youth’s height and weight were measured using a Seca 213 Portable Measuring Rod stadiometer and a Tanita BF697W Duo Scale. Participants were measured with their shoes removed, wearing light clothing. The measurements were taken twice, and a third measure was taken if the first two measures were more than 0.2 lb, or 0.25 in. different. Repeated measures were averaged. For participants who declined to have their height and weight measured, self-reported data was collected (self-reported weight: *n* = 3, and self-reported height: *n* = 1). BMI-for-age percentiles were calculated using height and weight and were compared to the age and sex-specific CDC BMI-for-Age growth charts [36].

### Development of youth (Individual) and household factors scales and scores

#### Youth food-related psychosocial scores

##### Nutrition knowledge

Fourteen questions assessed the level of food and nutrition knowledge related to breakfast food, food preparation, snacks, carryout food and beverages. See Additional file 1: Table S1 for description of psychosocial questions. Food knowledge scores were calculated by summing the number of correct responses for each youth (*n* = 285). The score ranged from 3 to 14 with a mean of 9.1 (SD = 2.5, α Cronbach = 0.62).

##### Intentions for healthy eating

Twelve questions focused on how respondents intend to select food for themselves in the future. The responses were graded by assigning 1 point to the healthiest option and zero otherwise. Scores ranged from 0 to 10 points with a mean of 3.1 (SD = 1.8, α Cronbach = 0.44). Higher healthy eating intention scores indicated that the respondent had a positive inclination towards healthy eating.

##### Food–related self-efficacy

Twelve self-efficacy questions were designed to assess the level of confidence respondents have in their ability to perform food-related behaviors such as the ability to select a healthy food or to cook in a certain way. The responses were based on options: “I know I can”, “I think I can”, “I am not sure I can”, and “I know I cannot”, and scored 3 to 0, respectively. Total scores ranged from 7 to 36 with a mean of 24.7 (SD = 3.8, α Cronbach = 0.68).

##### Nutrition outcome expectations

Eleven questions assessed a respondent’s expected health outcome from eating and drinking specific foods and beverages. Respondents were asked to choose whether a statement such as “I am more likely to get high blood pressure if I eat a lot of salty foods” was true, mostly true, mostly false, false or don’t know. We assigned 2 points for “true” responses, 1 point for “mostly true” and zero otherwise. Score ranged from 1 to 22 points, with a mean of 15.8 (SD = 3.65, α Cronbach = 0.61).

##### Youth food purchase frequency by venue

Youth were asked to report all the places they purchased food in the previous week. Participants were encouraged to list the food source name in each of the following categories: supermarket, corner store, convenience store, and fast-food/carry-out (see Additional file 2: Table S2). We then summed the purchase frequency of each store within the same food source category to calculate the total frequency of food purchasing by venue. We excluded 1 person who reported >100 corner store visits in one week.

##### Youth food preparation score

We asked youth that reported preparing food in the previous 7 days about their preparation methods. Each food preparation method was assigned the following score based on the healthiness of the method: fried = −1, baked = +1, microwaved = +1, raw = +1, other = 0 [30]. The total score for each cooking method was averaged and calculated taking into account the number of times food was prepared. The youth food preparation score ranged from −1 to +1, mean = 0.48 (SD = 0.6).

#### Household characteristics

##### Household food purchasing frequency by venue

Caregivers (*n* = 285) reported the number of times they purchased food from different food sources in the 30 days prior to the interview date. We provided a list of 18 different food sources (e.g. farmers’ market, urban farm, street vendor, public market, corner store, supermarket, carry-out, restaurant, and family/friends) (see Additional file 3: Table S3). For the purpose of our analysis, we selected the four main food sources used by our study sample, which were supermarkets (range from 0 to 30 visits; mean = 5.7, SD = 4.8), corner stores (range from 0 to 120 visits, mean = 8.4, SD = 16.9), convenience stores (range from 0 to 90 visits, mean = 3.5, SD = 8.9) and fast-food restaurants (range from 0 to 34, mean = 3.6, SD = 4.5). For each food source, food-purchasing frequency was treated as a continuous variable.

##### Household food preparation score

We asked caregivers to rank the top three most common cooking methods used when they prepared chicken, turkey (including ground turkey and turkey bacon), pork (including bacon), ground beef, fish, eggs, greens (excluding lettuce) and potatoes (Additional file 4: Table S4). The household food preparation score was calculated differently than the youth food preparation score as the nature of the question and the potential answers differed. Cooking methods were assigned scores as follow: deep fry or pan-fried with oil = −2; pan-fried, drained or use of cooking spray = −1; not prepared in the last 30 days = 0; pan-fried, drained and rinsed with hot water = +1; broiled/baked, grilled, steamed, boiled, eaten raw, or microwaved = +2. The household food preparation score was calculated by the weighted mean score for each food, taking into account the following proportion: 60 % (first method), 30 % (second method), and 10 % (third method). Then, the scores for all of 8 foods were summed to obtain the overall household food preparation score ranging from −1 to +2 (mean = −0.05; SD = 0.9).

#### Statistical analysis

Statistical analysis of the data was conducted using the software STATA 13.1 (College Station, TX, USA 2013). Two-tailed independent t-tests, ANOVAs and chi-squared tests were used to examine associations between groups of sex, age, income, and caregiver education level by servings of fruit, vegetable, and fiber intake. Ordered logit regression models were used to analyze the association between youth psychosocial characteristics and youth and household food behavior with FV and fiber intakes. Each model with the dietary outcomes (quartiles of FV and fiber intake) was regressed on different independent variables (intentions, self-efficacy, outcome expectancy, food knowledge, supermarket, corner store, convenience store, fast-food restaurant food purchase, food assistance, nutrition school program, and food preparation scores). The dietary outcomes of interest (fruit, vegetable and fiber intakes) were stratified by quartiles (presented in Additional file 5: Table S5), in which we interpret the increase in each quartile as a higher level of fruit, vegetable of fiber intake.

Assumptions of normality were investigated for each outcome of interest. Based on the Shapiro-Wilk test, the assumption of normality for FV and fiber intakes were violated. Thus, we log-transformed fruit and vegetable servings and the Shapiro-Wilk test still indicated that assumption of normality of error terms was likely violated. Moreover, we performed a sensitivity analysis to check whether the residuals of each regression were normally distributed by maintaining fruit and vegetable servings log transformed, and dietary fiber untransformed. Thus, we chose to perform an ordered logit model in order to obtain more power to detect changes among the different levels of intake, due to the non-linear fashion of our dietary data. The ordered logit model assumes that the effect of any of the independent variables should be the same regardless of the choice of level (quartiles) of fruit, vegetable, or fiber intake. We calculated the variance inflation factor for each model to check for collinearity by performing a multiple linear regression, which were all below 1.10. For all the models, we performed the specification error test to check whether our model had all the relevant predictors and if the linear combination of them was sufficient. In this test, the linear predicted value was statistically significant, and the linear predicted value squared was non-significant. The parallel assumption of the orderd logit regression was investigated by the likelihood-ratio test followed by the Brant Test, in which both tests failed to reject the null hypothesis that the coefficients were equal across categories. We also examined effect modification in all regression models by youth’s age, sex, and BMI. Since the interaction terms were not statistically significant they were excluded from the final models. All models were adjusted for youth’s age, sex, BMI percentile, total caloric intake and income ratio to 2014 federal poverty threshold as continuous variables. For all analyses, statistical significance has been defined by a *p*-value of < 0.05.

## Results

In total, 73.2 % of the youth interviewed consumed less than 2 servings a day of fruit and 76.2 % consumed less than 2.5 servings a day of vegetables. Table 1 displays the general characteristics of the participants stratified by fruit, vegetable and fiber intake. Fifty two percent of the youth were female, 53 % were 12 years or older. Most caregivers interviewed had a high-school degree (42 %) and a mean annual income between $20,000 and $30,000. Younger youth (10–11 years old) had a statically greater intake of vegetable servings when compared to older youth (12–14 years old). Interestingly, we also found that youth tend to have a statically higher intake of vegetable servings (mean = 1.9 servings/day) and fiber (mean = 16.1 g/day) during the school year than during the school break (mean = 1.4 servings and 12.9 g, respectively) (*p* = 0.04 and *p* = 0.03, respectively). Conversely, intake of fruit servings among low-income AA youth during summer break (mean = 1.6 servings/day) appeared to be higher than during the school year (mean = 1.1 servings/day) (*p* = 0.0002).

**Table 1:** Low-income urban African-American youth’s fruit, vegetable and fiber mean intake and frequency of youth meeting dietary recommendations by socio-demographic characteristics

		Food and nutrient intakes					
		Fruit, servings		Vegetables, servings		Total fiber (g)	
Individual characteristics of youth	n	Mean (SD)	RS^a^ (%)	Mean (SD)	RS^a^ (%)	Mean (SD)	RS^b^ (%)
Gender							
Male	130	1.4 (1.0)	21.5	1.6 (1.4)	20	14.4 (9.4)	7.5
Female	154	1.6 (1.2)	30.5	1.9 (1.5)	26	15.9 (11.8)	13.5
Age (years)							
10–11	126	1.6 (1.1)	28	2.0 (1.9)^c^	27	15.8 (11.2)	12
12–14	159	1.5 (1.2)	25	1.6 (1.5)^d^	20	14.8 (10.4)	10
Time of the Year							
School Year Intake	214	1.1 (0.8)^c^	21	1.9 (1.8)^c^	20	16.1 (11.4)^c^	10
Summer Break Intake	70	1.6 (1.2)^d^	5	1.4 (1.2)^d^	4	12.9 (8.7)^d^	2
Annual Income (US$)							
0–10,000	73	1.4 (0.9)	24.3	1.6 (1.7)	23.0	14.8 (9.9)	10.8
10,001–20,000	60	1.6 (1.3)	25.0	1.9 (1.7)	21.7	16.0 (11.6)	13.3
20,001–30,000	58	1.6 (1.1)	27.5	1.6 (1.4)	19.0	15.2 (10.5)	7.0
> 30,000	93	1.4 (1.2)	28.0	1.8 (1.8)	26.8	15.1 (11.0)	11.8
Caregiver Education Level							
< High School	104	1.4 (1.1)	24.1	1.6 (1.5)	19.2	14.9 (11.1)	11.5
High School	121	1.5 (1.6)	24.8	1.9 (1.8)	27.3	15.7 (11.1)	11.6
> High School	58	1.6 (1.3)	32.8	1.8 (1.8)	20.7	14.6 (9.0)	6.9
Food Assistance Participation							
WIC	248	1.55 (1.2)	23.9	1.81 (1.7)	20.4	15.4 (0.7)	10.5
SNAP	253	1.54 (1.2)	24.5	1.78 (1.7)	20.1	15.4 (11.2)	10.9
School Food Program Participation							
Breakfast	64	1.56 (1.1)	5.2	1.56 (1.6)	4.5	13.7 (8.2)	1.7
Lunch	216	1.51 (1.1)	19.7	1.77 (1.6)	19.3	15.4 (11.1)	9.1
Body Mass Index Classification							
Normal weight	153	1.54 (1.1)	27.9	1.80 (1.6)	25.9	15.8 (10.4)	13.0
Overweight	67	1.53 (1.3)	28.3	1.55 (1.6)	19.4	14.3 (10.3)	10.4
Obese	61	1.46 (1.2)	19.7	1.84 (1.9)	19.7	14.6 (12.6)	8.2

*Abbreviations*: *RS* recommended serving (% of youth meeting recommended consumption amount), *SD* standard deviation

^a^Frequency of youth meeting the recommended intake of fruit (2 servings per day) and vegetable (2.5 servings per day) according to US Dietary Guideline, 2010 [33]

^b^Frequency of youth meeting the Adequate Intake recommended by the DRI: female (≥26 g/day) and male (≥31 g/day)

^c,d^Are statistically different when comparing groups between the youth characteristics: 2-independent *t*-test; anova or chi-square

### Association between youth-level psychosocial variables and fruit, vegetable and fiber intakes

Fruit, vegetable, and fiber intake were positively related to youth intentions and self-efficacy for eating healthy (Table 2). In the adjusted model, an increase in intentions for healthy eating was associated with a 22 % (95 % CI: 1.06–1.41), 31 % (95 % CI: 1.15–1.51), and 46 % (95 % CI: 1.23–1.74) greater odds ratio for a higher intake of fruit, vegetable and fiber, respectively. Furthermore, an increase in self-efficacy was also associated with a 7 % (95 % CI: 1.03–1.12), 4 % (95 % CI: 1.01–1.09), and 10 % (95 % CI: 1.04–1.16), greater odds ratio for a higher intake of fruit, vegetable and fiber, respectively.

**Table 2:** Youth-level psychosocial determinants on fruit, vegetable, and fiber consumption among low-income AA youth^a,b^

Youth psychosocial determinants	Quartile of fruit intake (serving)	Quartile of vegetable intake (serving)	Quartile of fiber intake (grams)
	OR (95 % CI)	OR (95 % CI)	OR (95 % CI)
Outcome Expectancy	1.01 (0.94; 1.08)	1.01 (0.94; 1.07)	1.03 (0.91; 1.16)
Food-Knowledge	1.06 (0.95; 1.17)	1.02 (0.92; 1.13)	1.03 (0.91; 1.16)
Intentions	1.22 (1.06; 1.41)**	1.31 (1.15; 1.51)**	1.46 (1.23; 1.74)**
Self-Efficacy	1.07 (1.03; 1.12)**	1.04 (1.01; 1.09)*	1.10 (1.04; 1.16)**

Outcome expectancy range from 1 to 22 points; Nutrition Knowledge range from 3 to 14 points; Intentions for Healthy Eating range from 0 to 10 points; Food-related Self-efficacy range from 7 to 36 points

*p-value < 0.05; **p-value < 0.01

^a^Ordered Logit Regression Analysis on fruit, vegetable and dietary fiber daily servings

^b^Adjusted model for youth age, sex, youth BMI (percentile) and youth calorie intake, and estimated poverty threshold to income ratio

### Association between youth-level food behaviors and fruit, vegetable and fiber intakes

In an ordered logistic regression analysis controlling for estimated poverty threshold to income ratio, youth’s age, sex, BMI and calorie intake, youth receiving free/discounted breakfast had a higher odds ratio for fiber intake (OR = 2.75; 95 % CI: 1.10–6.90) (Table 3). We also found that an increase in youth purchasing for food at supermarkets was associated with an increase in vegetable serving and fiber intakes (OR = 1.26; 95 % CI: 1.06–1.50, OR = 1.28; 95 % CI: 1.03–1.58). No statistically significant associations were seen between food preparation behavior, and fruit, vegetable, and fiber intake. Total food purchasing frequency at the supermarket ranged from 0 to 14 (mean = 0.9; SD = 1.9); corner store ranged from 0 to 38 (mean = 3.3; SD = 4.7); convenience store ranged from 0 to 11 (mean = 0.6; SD = 1.4) and fast-food/carry-out ranged from 0 to 16 (mean = 1.5; SD = 2.7).

**Table 3:** Youth determinants of fruit and vegetable consumption among low-income AA youth^a,b^

Youth food behavior determinants	Quartile of fruit (Servings)	Quartile of vegetable (Servings)	Quartile of fiber intake (grams)
	OR (95 % CI)	OR (95 % CI)	OR (95 % CI)
Youth food purchasing frequency^c^			
Supermarket	1.08 (0.92; 1.28)	1.26 1.06; 1.50)**	1.28 (1.03; 1.58)*
Corner Store	1.04 (0.98; 1.10)	0.97 (0.92; 1.03)	1.01 (0.93; 1.08)
Convenience Store	1.03 (0.85; 1.25)	1.17 (0.95; 1.43)	1.22 (0.94; 1.59)
Fast Food	1.06 (0.93; 1.22)	1.11 (0.98; 1.26)	1.15 (0.95; 1.40)
Food Assistance^d^			
Breakfast at school	1.95 (0.92; 4.12)	1.14 (0.55; 2.38)	2.75 (1.10; 6.90)*
Lunch at school	1.24 (0.53; 2.89)	0.68 (0.31; 1.48)	1.33 (0.51; 3.51)
Youth Food Preparation Scale^g^	1.16 (0.69; 1.94)	1.21 (0.73; 1.99)	1.01 (0.55; 1.83)
Household food purchasing frequency^e^			
Supermarket	0.99 (0.93; 1.04)	0.99 (.94; 1.05)	0.96 (0.91; 1.03)
Corner Store	0.96 (0.92; 1.01)	0.97 (0.93; 1.02)	0.99 (0.93; 1.04)
Convenience Store	1.01 (0.95; 1.07)	1.00 (0.94; 1.05)	1.01 (0.94; 1.08)
Fast Food	1.00 (0.94; 1.06)	0.93 (0.88; 0.99)*	1.05 (0.97; 1.13)
Food Assistance^f^			
WIC	1.58 (0.88; 2.86)	0.78 (0.44; 1.40)	1.04 (0.53; 2.04)
SNAP	0.84 (0.42; 1.65)	1.50 (0.79; 2.85)	0.64 (0.29; 1.39)
Household Food Preparation Score^h^	1.09 (0.83; 1.42)	1.07 (0.83; 1.38)	0.99 (0.72; 1.36)

*p-value <0.05; **p-value < 0.01

^a^Ordered Logit Regression Analysis on fruit, vegetable and dietary fiber

^b^Controlled for youth’s age, sex, BMI (percentile), youth calorie intake, and estimated poverty threshold to income ratio

^c^Total frequency of youth’s purchase in the past 7 days (mean; min-max): supermarket (0.9; 0–29); corner store (3.3; 0–38); convenience store (0.61; 0–11); fast-food (1.5; 0–16)

^d^Free or discounted meal at school: reference (no breakfast at school, or no school meal at school)

^e^Total frequency of caregiver’s purchase in the past 30 days (mean; min-max): supermarket (3.8; 0–14); corner store (3.8; 0–29); convenience store (2.05; 0–28); fast-food (3.48; 0–25)

^f^Food assistance: reference = non-WIC or non-SNAP participants

^g^Youth Food Preparation Scale range from −1 to 1. (Mean 0.5 (SD 0.64)

^h^Household Food Preparation Scale range from −1 to +2 (Mean −0.05; SD 0.9)

### Association between household-level food related behaviors and youth’s fruit, vegetable and fiber intakes

Youth with parents who purchase food at fast-food restaurants showed a 7 % decrease in odds for vegetable intake (OR = 0.93; 95 % CI: 0.88–0.99) (Table 3). No statistically significant association was found between the household food preparation score or receiving food assistance such as SNAP or WIC, and fruit, vegetable, and fiber intakes.

## Discussion

This is one of the first studies to investigate the relationship between a youth’s dietary fruit, vegetable, and fiber intake with youth and household food-related behaviors in low-income AA communities in Baltimore City. Our findings indicate that increased scores for intentions and self-efficacy for healthy eating were positively associated with fruit, vegetable, and fiber intake. Free/discounted school breakfast and household and youth food acquisition frequency were associated with vegetable and fiber intake.

The majority of youth in our study failed to achieve the current recommendations for fruit, vegetable and fiber intake [37]. In this setting, participants had a lower intake of fruit servings when compared to the national levels [9], with only 26.8 % consuming at least 2 servings a day.

Youth’s psychosocial characteristics are thought to affect behavior through reciprocal interactions of personal factors, behavior and environment [38]. Self-efficacy, health outcome expectations, intention and knowledge are important psychosocial factors that might influence eating behavior. In our study, we found that intention and self-efficacy were important predictors of fruit, vegetable, and fiber intake. Self-efficacy levels were positively associated with fruit and vegetable consumption in African-American and Hispanic high-school students in Boston, USA [14]. A randomized controlled trial with first year undergraduate students from Australia found an increase in 0.83 servings of fruit and vegetable intake after one month exposed to materials targeting attitudes, self-efficacy, norms and intentions [39, 40]. Youth’s self-efficacy for FV consumption seems to mediate the negative relationship between parental barriers to purchasing healthy food items and youth’s fruit intake [18].

Another important determinant of FV intake in our study was having access to school meals. Youth who had either free or lower cost breakfast had higher intakes of fiber. In the US, the national school lunch program (NSLP) was established in 1946, but only recently, in 2011, school meal standards have become more closely aligned with dietary recommendations. The school food environment is an important factor associated with youth dietary intake. A study investigating the influence of the school food environment and dietary behavior in the US in 2003 found an inverse association between FV intake and à la carte programs, vending machines and availability of snacks in the schools [41]. We found a positive association between the School Breakfast Program and vegetable and fiber intake. Breakfast intake has also been associated with higher dietary fiber when compared to breakfast skippers in a sample of children and adolescents in the National Health and Nutrition Examination Survey 1999–2006 [42]. Contrary to some studies [43], we did not find an association between NSLP and FV and fiber intake. This may be explained by the fact that some school districts struggled to implement the new requirements during the 5-year implementation period [44]. Furthermore, during the period of our study, competitive foods were still available in the schools, as the Smart Snacks Rule was yet to be implemented in the 2014–2015 school year, which may influence a child’s dietary quality. Nevertheless, school meal programs were evidenced to be an important source of daily FV intake to low-income school-aged youth [45]. Hence, our findings are of great importance to support the newly passed legislation in Baltimore City in which free breakfast and lunch are offered to all youth regardless of their income [46].

Prior studies suggest that parental intake of fruits and/or vegetables is positively associated with youth intake of FV [17]. However, to our knowledge, no study has examined parental food acquisition behavior as a determinant of a youth’s fruit and vegetable consumption. We found that the youth of caregivers who frequently purchased food at a fast-food restaurant ate fewer vegetables per day than those whose caregivers shopped less often at fast-food restaurants, after controlling for youth’s age, sex, BMI, youth calorie intake, and income. This association may be due to the fact that parents who shop for food at fast-food stores may not prepare home meals for their children as often as those caregivers who shop less frequently at fast-food stores, and are therefore less likely to have fruits and vegetables available in the household. Caregivers have been purchasing food more frequently outside of their homes, while access to healthier items remains limited among minority populations. A study investigating the total energy intake in the household among low-income American families reported that 29 % of the caloric intake was derived from food eaten away from home [20]. Neighborhood availability and access to healthy outlets have been correlated with socioeconomic and racial disparities in the literature [47]. Even though a caregiver’s dietary behavior has been associated with a youth’s diet, little is known about a caregiver’s access to FV and its subsequent consumption among youth [48].

We found that an increased frequency of food purchases by youth at supermarkets or grocery stores was associated with higher intake of vegetable and fiber. Most youth (47 %) in our sample reported helping with food shopping for the household by going to the grocery store with the main food shopper. In our survey, we asked youth to only report the frequency of food shopping at different food sources when they were purchasing food for themselves (not including food that others purchased for them). In another manuscript using the same study population, youth reported that parents often supported their healthy eating behavior [49], which may help to explain our association between increased vegetable and fiber intake and grocery store shopping behavior. According to recent findings exploring BHCK data, peer support was not associated with dietary intake in our youth population [49]; therefore this factor was not included in our analysis. Other factors are known to influence consumption patterns, including affordability, quality, and in-store healthy food availability, which were not taken into consideration in our analysis and may explain the lack of association with fruit intake. Supermarkets are evidenced to have higher healthy food availability than other types of food stores, suggesting that availability of healthier items, such as FV, may be an important factor influencing diet [50]. Inaccessibility of healthy foods (e.g. fruits and vegetables) due to factors such as long distances to food stores from one’s household [51] and/or high prices [52] are important barriers that affect a youth’s diet behavior and food choices. A recent study found that increasing accessibility by decreasing distance to supermarkets was found to increase the odds of eating 4 servings or more FV in an urban American setting [53].

The present study has some limitations. First, this was a cross-sectional study, and therefore causal inferences cannot be made. In addition, our study focused on low-income AA urban population, which limits the transferability of results to other samples [54]. Second, our survey was administered to self-identified caregivers, under the assumption that they purchase most of the food and cook for their family members. However, some caregivers may not be the primary food purchasers for their households. Third, we only investigated the frequency of food purchased at various types of food venues, and did not take into consideration the quality or quantity of the acquired food. Future research should examine the influence of food quality, availability, and price at local food stores on youth dietary intake. For instance, inaccessibility to healthy foods (e.g. fruits and vegetables), due to factors such as long distances to food stores [51] and high prices [52], is an important barrier that may affect youth dietary and purchasing behavior. Fourth, correlations between psychosocial factors and FV intake should be interpreted with caution due to the low Cronbach’s alpha (intention for healthy eating <0.5). Fifth, we recognize that higher FV intake by itself might not affect body composition; however, low fruit, vegetable, and fiber consumption is associated with a higher intake of fat, sugar and salt [55]. Finally, due to the initial hypothesis of this study to focus only on youth fruit, vegetable, and fiber intake, we did not investigate factors associated with other food and beverage groups (e.g. whole grains, sugar-sweetened beverages, or snacks). However, encouraging FV and discouraging high-processed food may prevent obesity and promote health in the population.

## Conclusions

In summary, the results suggest that there is a relationship between youth psychosocial factors and youth- and household-level food-related behaviors, on fruit, vegetable, and fiber intake in youth living in low-income, urban food deserts. National nutrition programs aiming to improve nutrition and food security were positively associated with children’s FV intake. Future public health programs, interventions and policies should consider targeting the factors associated with fruit and vegetable intake presented in this manuscript to increase healthy eating behaviors among youth. Future research should attempt to identify further barriers to and facilitators of access to FV, and other risk factors associated with high-processed food to better understand ways to improve youth dietary quality and combat childhood obesity.

## Additional files

Additional file 1: Table S1. Food-related psychosocial factors in CIQ. (DOCX 22 kb) Additional file 2: Table S2. Food Purchasing Frequency by Venue in CIQ. (DOCX 18 kb) Additional file 3: Table S3. Food Purchasing Frequency by Venue in AIQ. (DOCX 18 kb) Additional file 4: Table S4. Food Preparation Methods in AIQ. (DOCX 18 kb) Additional file 5: Table S5. Fruit serving, vegetable serving and fiber intake stratified by quartiles. (DOCX 20 kb)
