# Supplementary material for: Household, psychosocial, and individual-level factors associated with fruit, vegetable, and fiber intake among low-income urban African American youth
Source: BMC Public Health. 2016 Aug 24;16(1):872. doi: 10.1186/s12889-016-3499-6 (PMC4997673; doi:10.1186/s12889-016-3499-6)
Supplement: Additional file 1: Table S1. — Food-related psychosocial factors in CIQ. (DOCX 22 kb) [file 12889_2016_3499_MOESM1_ESM.docx]

**Supplementary material**

| ***Table S1.*** *Food-related psychosocial factors in CIQ* | |
| --- | --- |
| **Food-Related Knowledge** | 1. Which of the following adds the least amount of fat? (Froot Loops; Rice Krispie; Honey Nut Cheerios; Don’t know) 2. Which breakfast has less fat? (Oatmeal with fruit; An omelet with bacon; Poptarts; Don’t know) 3. Which breakfast cereal do you think has more fiber? (Raisin Bran; Lucky Charms; Frosted Flakes; Don’t know) 4. What is the healthiest way to eat vegetables? (Baby carrots with low fat dip; Greens cooked with added butter; Hash browned potatoes fried in a pan; Don’t know) 5. What’s the healthiest spread to put on a sandwich? (Butter; Mayonnaise; Mustard; Don’t know) 6. Which snack has less sugar? (Tasty cake; Cookie; Granola Bar; Don’t know) 7. Which snack has less salt? (Pretzels; Baby Carrots; Hot Cheetos; Don’t know) 8. Which potato chip has less fat? (Regular Utz potato chips; Doritos; Baked Utz potato chips) 9. Which sandwich bread is healthier? (100% whole wheat; White bread; Potato Bread; Don’t know) 10. Which fast food has less fat? (Chinese Egg Roll; Chicken box; Turkey Sub; Don’t know) 11. Which side is lowest in fat? (French Fries; Cooked greens; Chips; Don’t know) 12. Which soda has less sugar? (Grape soda; Coke; Coke zero; Don’t know) 13. Which drink has less sugar? (Energy drink; fruit-flavored water; Diet half-and-half; Don’t know) 14. Which milk has less fat? ( Whole milk, Skim milk; 2% milk; Don’t know) |
| **Food Intentions** | 1. If you wanted a snack, which would you pick? (Potato chips; Pretzels; Yogurt) 2. The next time you are thirsty, which would you choose? (Regular soda; Fruit-flavored water; Plain water) 3. If you had to eat cereal, which would you choose? (Kix; Life cereal; Froot Loops) 4. The next time you want an after-school snack, which would you choose? (Sunflower seeds, French fries; Candy) 5. If you had to eat at a fast-food restaurant or carryout, which meal would you choose? (Burger; Turkey sandwich; Fried chicken) 6. If you had to eat a vegetable, which would you choose? (Baby carrots; Corn; Potatoes) 7. If you had to drink a fruit beverage, which would you choose? (Sugar-free drink mix; Fruit punch; Fruit flavored soda) 8. If you had to choose a fruit snack, which would you choose? (Apple with caramel dip; Grapes; Fruit roll-up) 9. If you had to put something on a sandwich, which would you choose? (Mustard; regular mayonnaise; Butter) 10. If you had to drink milk, which would you choose (include Lactaid)? (Regular Whole milk; 2% milk; 1% or skim milk) 11. If you had to eat a quick breakfast, what would you choose? (Poptarts; None – I’d skip breakfast; Piece of fruit) 12. If you were making a sandwich, what type of bread would you choose? (White Bread; Potato bread; 100% Whole wheat/grain bread) |
| **Food-Related Self-Efficacy** | How sure you are that you can do it, given your daily life:   1. Eat vegetables several times a day? 2. Reduce the amount of potato chips to only one small bag a day? 3. Eat a bowl of low-sugar cereal for breakfast, even when running late for school? 4. Drink sugar-free drinks instead of fruit punch? 5. Choose vegetables for a snack instead of potato chips or snack cakes? 6. Eat at least one fruit everyday outside of school? 7. Ask for low-fat mayonnaise or miracle whip on my sandwich? 8. Buy fruit to snack on at the corner store? 9. Buy baked chips instead of regular chips at the corner store? 10. Try healthier side dishes at the fast food restaurants like having apples or yogurt instead of fries? 11. Talk to parents about buying healthy snacks? 12. Make a sandwich on 100% whole wheat bread versus white bread? |
| **Outcome Expectancies** | 1. I would be healthier if I ate French fries three times a week instead of eating French fries seven days a week. 2. I would lose weight if I drink diet soda instead of regular soda. 3. I am more likely to get heart disease if I eat fried chicken instead of baked chicken. 4. I am more likely to get high blood pressure if I eat a lot of salty foods. 5. I will gain weight if I eat a lot of fatty foods (like potato chips). 6. I would have more energy if I ate more fruits and vegetables. 7. I will get diabetes if I eat a lot of sugary foods (like tasty cakes and ice cream). 8. I would have more energy to exercise or play sports if I ate more whole grains 9. I would feel better if I drank more water and less soda 10. I would feel better if I ate more fiber 11. I would be less likely to gain weight if I added less butter to my food |
|  |  |
