# Supplementary material for: Household, psychosocial, and individual-level factors associated with fruit, vegetable, and fiber intake among low-income urban African American youth
Source: BMC Public Health. 2016 Aug 24;16(1):872. doi: 10.1186/s12889-016-3499-6 (PMC4997673; doi:10.1186/s12889-016-3499-6)
Supplement: Additional file 2: Table S2. — Food Purchasing Frequency by Venue in CIQ. (DOCX 18 kb) [file 12889_2016_3499_MOESM2_ESM.docx]

**Supplementary material**

| ***Table S2.*** *Food Purchasing Frequency by Venue in CIQ* |
| --- |
| Think about all places you bought food during the past 7 days, What are all places that you shop in each category? I am interested in times when you spend money on food for yourself:   1. Supermarket/Grocery Store 2. Corner Store 3. Convenience Store 4. Fast-food restaurant/ carryout 5. School/ recreation center 6. Other (food truck, arabber, drug store) |
