# Supplementary material for: Household, psychosocial, and individual-level factors associated with fruit, vegetable, and fiber intake among low-income urban African American youth
Source: BMC Public Health. 2016 Aug 24;16(1):872. doi: 10.1186/s12889-016-3499-6 (PMC4997673; doi:10.1186/s12889-016-3499-6)
Supplement: Additional file 3: Table S3. — Food Purchasing Frequency by Venue in AIQ. (DOCX 18 kb) [file 12889_2016_3499_MOESM3_ESM.docx]

**Supplementary material**

| ***Table S3.*** *Food Purchasing Frequency by Venue in AIQ* |
| --- |
| For each source location, please tell me how many times in the last 30 days you got/purchased food:   1. A farmer’s market in Baltimore City 2. A local or urban farm stand 3. An arabber or mobile produce cart 4. Street vendor 5. A public market 6. The virtual supermarket program 7. A local corner store 8. A supermarket or grocery store 9. A wholesale food store 10. A local carryout 11. A chain fast-food restaurant 12. A specialty store (bakery, African store, coffee store) 13. A sit-down restaurant, bar/pub 14. Food pantry 15. Church community center 16. Convenience store 17. Family/friends 18. Other |
