# Supplementary material for: Household, psychosocial, and individual-level factors associated with fruit, vegetable, and fiber intake among low-income urban African American youth
Source: BMC Public Health. 2016 Aug 24;16(1):872. doi: 10.1186/s12889-016-3499-6 (PMC4997673; doi:10.1186/s12889-016-3499-6)
Supplement: Additional file 4: Table S4. — Food Preparation Methods in AIQ. (DOCX 18 kb) [file 12889_2016_3499_MOESM4_ESM.docx]

**Supplementary material**

| ***Table S4.*** *Food Preparation Methods in AIQ* |
| --- |
| Below is a list of foods you may have prepared for your household in the past 30 days. For each food item, I would like to know what top 3 most common cooking methods were when it was cooked in your home. Please tell me whether you: 1) deep fried; 2) pan-fried with oil; 3) pain-fried drained; 4) pain-fried, drained, and rinsed with hot water; 5) used cooking spray; 6) broiled/baked; 7) grilled or BBQed; 8) steamed; 9) boiled; 10) raw; 11) microwaved:   1. Chicken 2. Pork (include bacon) 3. Ground beef 4. Turkey (include ground turkey or bacon) 5. Eggs 6. Greens (not lettuce) 7. Potatoes; 8. Fish |
