# Supplementary material for: Household, psychosocial, and individual-level factors associated with fruit, vegetable, and fiber intake among low-income urban African American youth
Source: BMC Public Health. 2016 Aug 24;16(1):872. doi: 10.1186/s12889-016-3499-6 (PMC4997673; doi:10.1186/s12889-016-3499-6)
Supplement: Additional file 5: Table S5. — Fruit serving, vegetable serving and fiber intake stratified by quartiles. (DOCX 20 kb) [file 12889_2016_3499_MOESM5_ESM.docx]

**Supplementary material**

Table S5: Fruit serving, vegetable serving and fiber intake stratified by quartiles.

| Food/Nutrient Intakes | n | | Median | Range (min – max) | | |  |
| --- | --- | --- | --- | --- | --- | --- | --- |
| Fruit serving intake |  | |  |  |  |  |  |
| Q1 | 74 | | 0.43 | 0 – 0.71 | |  |  |
| Q2 | 73 | | 1.07 | 0.75 – 1.29 | |  |  |
| Q3 | 67 | | 1.57 | 1.32 – 2.04 | |  |  |
| Q4 | 70 | | 2.8 | 2.07 – 6.5 | |  |  |
| Vegetable serving Intake |  | |  |  | |  |  |
| Q1 | 69 | 0.30 | | 0 – 0.54 | | | |
| Q2 | 72 | | 0.90 | 0.55 – 1.25 | |  |  |
| Q3 | 74 | | 1.74 | 1.26 – 2.35 | |  |  |
| Q4 | 69 | | 3.56 | 2.36 – 9.25 | |  |  |
| Dietary Fiber Intake (grams) |  | |  |  | |  |  |
| Q1 | 69 | | 5.94 | 2.37 – 7.67 | |  |  |
| Q2 | 72 | | 9.72 | 7.68 – 12.12 | |  |  |
| Q3 | 74 | | 15.96 | 12.15 – 18.89 | |  |  |
| Q4 | 70 | | 26.70 | 18.96 – 62.98 | |  |  |

Note: The increase in each quartile is interpreted as a higher level of fruit, vegetable of fiber intake.
